# Effect of Addition of Cetylpyridinium Chloride Cationic Surfactant on the Antimicrobial Activity of Chlorhexidine Endodontic Irrigant

**DOI:** 10.1155/2024/2449447

**Published:** 2024-04-05

**Authors:** Hiba A. Al-Sada, Hikmet A. Al-Gharrawi

**Affiliations:** ^1^Department of Conservative Dentistry, College of Dentistry, Mustansiriyah University, Baghdad 10001, Iraq; ^2^Health Center Al-Makaseb, Al-Amel Sector, Baghdad Al-Karkh Health Department, Iraqi Ministry of Health, Baghdad, Iraq

## Abstract

**Background:**

Endodontic irrigants are essential for disinfecting the root canal system. None of the currently available irrigants perfume sufficiently. However, most products contain surfactants, which enhance the antimicrobial properties of the irrigants.

**Objectives:**

To evaluate the effect of cetylpyridinium chloride (CPC) surfactant on the antimicrobial activity of chlorhexidine and compare it with that of chlorhexidine (CHX) and Biopure MTAD against *Enterococcus faecalis*, *Staphylococcus aureus*, and *Candida albicans*.

**Materials and Methods:**

In this in vitro study, three microorganisms were used (*E. faecalis*, *S. aureus*, and *C. albicans*), and each organism was treated with three different irrigants: 2% CHX, 2% CHX + 0.2% CPC, and 100% Biopure MTAD. The antimicrobial activity was evaluated by direct contact assay for 5 min of contact time. The colony-forming unit per mL was calculated after antimicrobial treatment and 24 hr of incubation at 37°C. The data were statistically analyzed using Statistical Package for Social Science (SPSS) version 22. The Kruskal–Wallis and the multiple Wilcoxon sum rank tests were used. The level of significance was set at 0.05.

**Results:**

The result showed a nonsignificant difference between the different irrigants against *E. faecalis*. Among *S. aureus* subgroups, 2% CHX was statistically significant and more efficient than MTAD. Among *C. albicans* subgroups, 2% CHX and combined irrigant (2% CHX + 0.2% CPC) were statistically more efficient than MTAD. The 2% CHX and combined irrigants were equally effective against all the tested microorganisms.

**Conclusions:**

All the used irrigants have comparable effects against *E. faecalis* after 5 min. CHX have a comparable effects to that of the combined irrigant and more efficient against *S. aureus* than MTAD. CHX and the combined irrigant have potent antimicrobial activity against *C. albicans* superior to MTAD. CPC surfactant can be used with CHX to overcome its clinical drawbacks or limitations without altering or reducing its antimicrobial activity.

## 1. Introduction

The main goal of the endodontic therapy is to provide a biologically suitable condition inside the root canal system that permits healing and ongoing maintenance of the periradicular tissue's health [1]. Therefore, complete debridement and effective elimination of microbiological irritants, including microorganisms and their byproducts, followed by obturation of the entire root canal system, are necessary for successful endodontic therapy [2].

The complicated internal anatomy of the root canals, such as the isthmus, lateral canals, accessory canals, creates a favorable habitat for microorganisms to live and conduct their pathogenic processes. Some studies have found that these regions in the root canal system are left untouched during mechanical instrumentation [3–6], which makes the goal of endodontic therapy unlikely to be achieved with mechanical instrumentation alone. As a result, chemical irrigants are essential for disinfecting these complicated root canal anatomies [7]. An ideal irrigant should have strong antibacterial properties, dissolve any remaining pulp tissues without posing systemic health risks, and be readily available [8].

Some microorganisms such as *Enterococcus faecalis*, *Staphylococcus aureus*, and *Candida albicans* are the primary causative agents responsible for the development of necrotic pulp and periapical pathosis [9, 10]. These microorganisms can resist the chemomechanical preparation and cause failure in root canal therapy [10, 11].

Chlorhexidine (CHX) is one of the most commonly used and investigated root canal irrigants in endodontics. CHX has broad-spectrum antimicrobial activity and is an effective irrigant against gram-positive and gram-negative oral pathogens, yeasts, and fungi. CHX is available in liquid and gel form and is used frequently at concentrations of 0.2%–2% [10]. One of the drawbacks of CHX is the high surface tension that affects its penetration to complex root anatomy, reducing its antimicrobial effect. Studies revealed that this drawback could be overcome by incorporating surface-active agents, such as surfactants in the irrigant composition [12, 13].

Several commercial irrigants that contain modifiers or surfactants are being touted as having more antimicrobial activities than their traditional counterparts. The Biopure MTAD (Tulsa, Dentsply) is a mixture of doxycycline, citric acid, and polysorbate 80 (surfactant). It is only applied as a final rinse in conjunction with NaOCl. Although most research has reported that MTAD is preferable to CHX, controversial studies contend that no such distinction exists [14–18]. The nonavailability of MTAD and other commercial products has forced researchers to create a replacement by creating locally produced irrigants, which would be inexpensive and readily available for use in endodontic procedures.

Cetylpyridinium chloride (CPC) is a quaternary ammonium compound and cationic surfactant that exhibits broad-spectrum antimicrobial activity against gram-positive pathogens, fungi, and yeasts. It has antiseptic qualities and is frequently used as an active compound in toothpaste, mouthwashes, lozenges, or mouth sprays for treating mild mouth and throat infections [19].

Thirunarayanan and Hegde [13] were the first to report the use of CPC as a surfactant in combination with CHX for the cleaning of root canals. They found that adding CPC improved the depth of CHX penetration by lowering the irrigant's surface tension. The previous study only assessed the depth of penetration of the combined irrigant. Still, there is a need to investigate the antimicrobial activity of this new combination. To date, no previous study has been conducted to assess the effect of CPC on the antimicrobial activity of CHX.

This study evaluated the antimicrobial activity of the combination of CHX + CPC irrigant and compared it with that of CHX and MTAD on *E. faecalis*, *S. aureus*, and *C. albicans* using direct contact assay. We hypothesized that there was no substantial difference in the antimicrobial activity of various irrigants.

## 2. Materials and Methods

### 2.1. Irrigant Solutions

Three irrigant solutions were used in this study: 2% CHX (Stalowa Wole, Poland), combined irrigant (2% CHX + 0.2% CPC) prepared in the laboratory by dissolving 0.2 g of CPC powder (Sigma–Aldrich, Schnelldorf, Germany) in 100 mL of a 2% CHX solution and mixed well until the powder dissolved completely to reach final irrigant concentrations of 2% CHX + 0.2% CPC (w/v) [20], and Biopure MTAD (Tulsa, Dentsply) which was prepared according to the manufacturer's instructions. All prepared irrigants were filtered using a 0.22-*µ*m Millipore syringe filter to ensure sterilization and stored in sterile test tubes at room temperature.

### 2.2. Microbial Strain and Standardization

Pure cultures of *E. faecalis*, *S. aureus*, and *C. albicans* grown on brain heart infusion (BHI, Liofilchem, Italy) agar plates were obtained from the Educational Labs of the Ministry of Health, Baghdad, Iraq. Grown colonies were identified according to their morphology and Gram stain. Further identification was based on biochemical tests, which were carried out depending on the VITEK 2 System (Biomerieux, France). Isolated colonies of these microorganisms were suspended in a sterile saline (0.9% NaCl) solution after being grown aerobically in BHI broth for 24 hr. The cell suspension was adjusted spectrophotometrically at 600 nm to match the turbidity of 0.5 McFarland scale (1.5 × 10^8^ colony-forming units (CFU)/mL) [1, 21].

### 2.3. Direct Contact Assay

This in vitro study was carried out at the educational laboratories of the College of Health and Medical Technologies, Baghdad. One milliliter of each microbial suspension was obtained and added to a tube containing 1 mL of the irrigant solutions (2% CHX, 0.2% CPC + 2% CHX, and 100% MTAD) for the experimental time of 5 min [1, 21]. After the indicated exposure time, 100 *µ*L samples were obtained, added to 900 *µ*L of sterile saline contained in Eppendorf tubes, and serially diluted. A vortex mixer (Gemmy Industrial Crop, Taipei, Taiwan) was used throughout the procedure to uniformly mix the test tube contents. Finally, droplets of 10 *µ*L from the diluted and undiluted tubes were cultured on Mueller–Hinton agar plates and incubated at 37°C for 24 hr for colony counting under magnification using a colony-counting device (Suntex, Taiwan). The procedure was performed ten times for each subgroup [1, 21, 22].

The CFU/mL of culture was calculated as follows [22].

(1) $$\begin{array}{c} \text{CFU/mL}=\frac{\text{Number of colonies}\times \text{dilution factors}}{\text{Volume of culture plate}}. \end{array}$$

### 2.4. Statistical Analysis

Data description, analysis, and presentation were performed using Statistical Package for Social Science (SPSS version 22, Chicago, Illinois, USA). Shapiro–Wilk test findings showed that the data were not normally distributed; therefore, the Kruskal–Wallis test was used to determine the presence of a significant difference in the mean rank among groups. A multiple Wilcoxon sum rank test was used for multiple comparisons between groups adjusted by the Dunn–Bonferroni method. The *p*-value was set at 0.05.

## 3. Results

**Figure 1:**
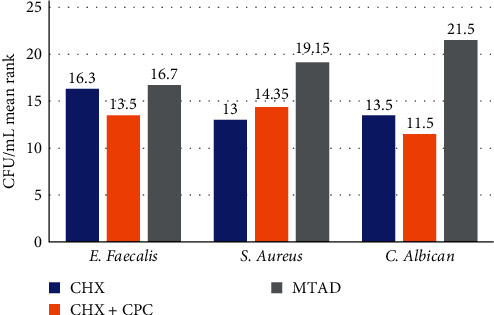
Bar chart for the CFU/mL mean rank of all groups. CHX + CPC has the lowest CFU/mL mean ranks among *E. faecalis* and *C. albicans* subgroups. CHX has the lowest CFU/mL mean rank among *S. aureus* subgroups. MTAD has the highest CFU/mL mean rank among all groups. CHX, chlorhexidine; CPC, cetylpyridinium chloride.

The mean rank of CFU/mL values of all groups is shown in Figure 1. The Kruskal–Wallis test revealed that there was no significant difference in the antimicrobial activity of CHX, CHX + CPC, and MTAD on *E. faecalis* (*p*=0.325), while there was a significant difference in the antimicrobial activity of these irrigant solutions on *S. aureus* (*p*=0.041) and *C. albicans* (*p*=0.008; Table 1).

When the intergroup comparison was made among *S. aureus* subgroups, only the antimicrobial activity of 2% CHX was found to be statistically significant (*p*=0.016) compared to that of MTAD. Although there was no statistically significant difference in the antimicrobial activity between CHX + CPC and MTAD on *S. aureus* (*p*=0.060), there was medium practical significance with an effect size of *F* = 0.42 (small < 0.3, medium = 0.3–0.499, and large ≥ 0.5) [23]. In contrast, in the *C. albicans* subgroup, both CHX and CHX + CPC antimicrobial activities were statistically significant compared to that of MTAD (*p*=0.019 and 0.003), while there were no significant differences between that of CHX and CHX + CPC groups (see Table 2).

## 4. Discussion

Successful endodontic treatment is achieved by completely eradicating microorganisms responsible for pulpal and periradicular infections. Mechanical instrumentation, chemical irrigants' flushing, and antimicrobial activity typically eliminate microorganisms from the root canal [6]. None of the currently available irrigants perform satisfactorily. Previously, several combined products became commercially available for root canal irrigation. These products contain antibacterial compounds or surfactants added to the primary irrigant to enhance their antimicrobial activity and improve clinical performance when added [24]. Therefore, it is necessary to study the effect of these new additives on the antimicrobial activity of primary irrigants.

Endodontic infections are polymicrobial. *E. faecalis*, *S. aureus*, and *C. albicans* were selected in this study because these microorganisms are frequently identified in endodontic infections and have a higher resistance to that of the chemomechanical preparation when compared with that of other microbial species, suggesting that they comprise leading causes of the failure of endodontic treatment [10].

Despite the belief that using the biofilm mode of microbial growth could reproduce a better picture of the microbial organization to preciselymimic an in vivo state, planktonic cultures have still been widely used to determine the antimicrobial effect of root canal irrigants [1, 25–28].

A VITEK 2 system was used for the identification of the used microorganisms because it is an easy-to-use system that provides fast (4–15 hr), more accurate, and highly reproducible results [29]. In addition, it can avoid unintentional cross-contamination or environmental contamination because it is a closed system. Many specimens can be automatically controlled simultaneously using the VITEK 2 system [30].

The microbial suspension was adjusted to 0.5 McFarland by spectrophotometer at a wavelength of 600 nm because this is a known wavelength that minimizes cell damage and growth and is not destructive [11].

The combination of 2% CHX + 0.2% CPC was used as an endodontic irrigant for the first time by Thirunarayanan and Hegde [13] to overcome one of the CHX irrigant drawbacks, which is the high surface tension. The antimicrobial activity of this combination was assessed as mouthwash and in different concentrations. However, there is still a need to investigate the antimicrobial activity of this new combination as an endodontic irrigant and compare it with that of CHX irrigant alone without any additions. Biopure MTAD was also used in this study because there was a controversy about the antimicrobial effect of MTAD compared to that of CHX.

In this study, direct contact assay was used despite not being able to represent the clinical situations accurately seen in endodontic infections; it gives some insights and allows comparison of the chemicals because it is a quantitative, reproducible method unaffected by the antimicrobial agent's solubility and diffusibility [11, 31]. This method measures the effect of direct and close contact between a fixed volume of the antimicrobial agent and a fixed volume of a microbial suspension for a particular time [2]. The antimicrobial activities of the used irrigants in this study were assessed after 5 min because MTAD, according to the manufacturer's instructions, requires a contact time of 5 min [1].

This study showed that all the used irrigants (CHX, CPC + CHX, and MTAD) were equally effective against *E. faecalis*, and there was no difference in their antimicrobial activity. Portenier et al. [32] observed similar outcomes when they compared the antibacterial activity of MTAD to that of CHX. In another study, Mohammadi [33] stated that CHX and MTAD were equally effective against the planktonic form of *E. faecalis*.

Contrary to the earlier-stated result, the findings of this study was not consistent with Davis et al. [15] and Mattigatti et al. [34] who stated that MTAD showed more zones of inhibition and antimicrobial activity on *E. faecalis* than those of 2% CHX; this may be related to the differences in the methodology used for assessing the antimicrobial activities of the irrigants used in their studies for the agar diffusion test that is affected by the irrigant diffusibility, which is in turn affected by the irrigant surface tension [15, 34]. MTAD has less surface tension than 2% CHX because of the presence of surfactant in its composition; therefore, it had more inhibition zones [13]. The present study was consistent with that of Agrawal et al. [17], who stated that MTAD is more effective than 2% CHX, which may be related to the differences in the contact time between the microorganism and the irrigants. They assessed the antimicrobial activity immediately after irrigation and after 48 hr.

Regarding *S. aureus*, CHX showed more antimicrobial activity than that of MTAD. Although the results showed that there was no statistical difference between the antimicrobial activities of CPC + CHX and MTAD, implying that CPC may adversely affect the antimicrobial activity of CHX against *S. aureus*, there was no statistical difference in the antimicrobial activities of CPC + CHX and CHX. Both groups demonstrated comparable efficacy. However, the practical significance has been carried out and represented by the effect size, which showed that CPC + CHX has more practical importance or impact than MTAD, which may be related to the differences in the mechanisms of action of the irrigants. The results were consistent with Mattigatti et al. [34], who showed that 2% CHX has a higher inhibitory effect on *S. aureus* than that of MTAD and other irrigants used in their study. However, this result was inconsistent with that of Chandrappa et al. [35], who found that MTAD possesses superior antimicrobial activity against *S. aureus* when compared with CHX antimicrobial activity, which may be related to methodological differences. They used CHX and MTAD to disinfect the gutta-percha by immersing the cones in the irrigant solutions for 30 s, 1 min, and 5 min, separately. Then, the disinfected cones were incubated in a medium for 7 days.

Regarding *C. albicans*, both CHX and CPC + CHX showed more efficient antimicrobial activity than that of MTAD, which may be related to the composition of MTAD because its antimicrobial activity is related to its doxycycline component, a bacteriostatic antibiotic; MTAD has less antimicrobial activity against *C. albicans* than that of CHX and the combined irrigant CHX + CPC. This result was consistent with several studies. Ruff et al. [14] showed that 2% CHX has a higher antimicrobial activity against *C. albicans* than that of MTAD. Mattigatti et al. [34] showed that 2% CHX has a higher inhibitory effect on *C. albicans* than that of MTAD and other irrigants used in their study. Misuriya et al. [36] showed that MTAD was less effective against *C. albicans* than 2% CHX.

The study results showed no differences in the antimicrobial activity of CHX and CPC + CHX against the microbial species used; therefore, CPC does not cause any alteration or reduction in the antimicrobial activity of CHX. However, it may increase the CHX antimicrobial activity by lowering CHX surface tension, increasing the penetration depth into the dentinal tubule; reaching this area that cannot be achieved with CHX alone, so further research is needed to cover this area. No previous study compared the antimicrobial activity of CHX and CPC + CHX as endodontic irrigants on the microorganisms used. There is a study assessing the antimicrobial activity of CPC alone as an endodontic irrigant. Estrela et al. [37] suggested that 0.2% CPC alone showed the same antimicrobial activity as 2% CHX in their investigation to confirm the antimicrobial activity of CPC in *E. faecalis* infected root canals. Becker et al. [38] compared the antimicrobial activities of CHX and CHX + CPC as mouthwashes on oral biofilms, and no differences were found between the CHX + CPC and CHX groups in all experimental conditions; the present results indicate that both groups demonstrated comparable efficacy. However, the combination of CHX and CPC was used in different concentrations, as with the mouthwash, and showed a synergistic effect, increasing the overall antimicrobial activity [39]. The differences in this result may be related to assessing the antimicrobial activity of CHX and CHX + CPC on different microorganisms using different methods.

All the irrigants used showed efficient antimicrobial activity against *E. faecalis*, *S. aureus*, and *C. albicans*. The antimicrobial activity of CHX is related to its structure as a cationic molecule that binds to negatively charged phospholipid and lipopolysaccharide molecules on the microbial cell wall. Chlorhexidine induces cytolysis-mediated cell death at high concentrations. It causes changes in the protein structure of the cell (precipitation or coagulation of cytoplasmic proteins) and the release of critical intracellular components, including nucleotides [40]. Cetylpyridinium chloride antimicrobial activity is related to its cationic portion, which promotes the connection with anionic compounds at the microbial cell wall, resulting in the alteration of the integrity of the cytoplasmic membrane, inhibition of cellular functions, and induction of cell death [41]. The antimicrobial effect of MTAD may be primarily attributed to the doxycycline component of the irrigant, a broad-spectrum antibiotic effective against a wide range of microorganisms. Doxycycline inhibits protein synthesis and reversibly binds to the 30s ribosomal subunits of susceptible microorganisms [18].

The presence of multiple microorganisms in biofilm form in the infected root canal may mean that an irrigant that works efficiently against a single organism in vitro may not work as well against the same organism in vivo. Further clinical trials or using more complex environments such as tooth models are necessary to corroborate the in vitro study findings regarding the efficacy of the irrigants against endodontic microorganisms. The cytocompatibility of this new combination should also be investigated.

## 5. Conclusion

All the irrigants used have comparable effects against *E. faecalis* after 5 min of contact time. CHX alone has more efficient activity than that of MTAD and an equivalent effect to the combined irrigant against *S. aureus*. CHX and the combined irrigant (CPC + CHX) have potent antimicrobial activity against *C. albicans*, which is more efficient than MTAD. CPC surfactant can be used with CHX to overcome some clinical drawbacks or limitations without altering or reducing its antimicrobial activity.

## Figures and Tables

**Table 1:** Kruskal–Wallis test analysis of the CFU/mL mean ranks of all groups.

Microorganisms		CHX	CHX + CPC	MTAD	Kruskal–Wallis	*p*-Value
*E. faecalis*	Mean rank	16.30	13.50	16.70	2.248	0.325
*S. aureus*	Mean rank	13.00	14.35	19.15	6.399	**0.041**
*C. albicans*	Mean rank	13.50	11.50	21.50	9.704	**0.008**

*Note*: The Kruskal–Willis test revealed that there was a significant difference in CFU/mL mean ranks among *S. aureus* and *C. albicans* subgroups as *p*  ≤ 0.05. CHX, chlorhexidine; CPC, cetylpyridinium chloride. Bold values illustrate a significant difference between tested subgroups *p* < 0.05.

**Table 2:** Multiple Wilcoxon sum rank test for multiple comparisons of CFU/mL mean ranks among *S. aureus* and *C. albicans* subgroups.

Microorganism	Groups		*Z* test	*p*-Value
*S. aureus*	CHX	CHX + CPC MTAD	0.528	0.597
			2.406	**0.016**
	CHX + CPC	MTAD	1.878	0.060

*C. albicans*	CHX	CHX + CPC MTAD	0.589	0.556
			2.944	**0.019**
	CHX + CPC	MTAD	2.355	**0.003**

*Note*: Bold values illustrate a significant difference between tested subgroups. CHX, chlorhexidine; CPC, cetylpyridinium chloride.

## Data Availability

The data supporting the findings of the study are available from the corresponding author upon reasonable request.
